# Memory B Cells, the HLA-B*57 Allele and Natural Control of HIV Infection

**DOI:** 10.1016/j.ebiom.2017.06.008

**Published:** 2017-06-13

**Authors:** Rob J. Center

**Affiliations:** Burnet Institute, Disease Elimination Program, Life Sciences, Melbourne, Victoria, Australia

In a small number of HIV-1 infected individuals natural restriction of viremia to very low or undetectable levels is achieved without the use of combined antiretroviral therapy (cART). Identifying the factors underpinning viral suppression in these so-called elite controllers (EC) has long been a goal with the aim of informing potential therapeutic and infection prevention strategies. A number of viral and host factors associated with EC have been identified, implying that viral control within EC as a whole and probably within at least some individual EC is multifactorial. Host HIV-specific cellular immune responses that have been associated with EC include potent Gag-specific CD8 + T cells, especially when associated with expression of the HLA-B*57 allele (Migueles et al., 2000) and CD4 + T cell responses. The role of humoral immunity is less clear. It was reported that EC had elevated polyfunctional HIV-specific non-neutralizing antibody responses in the absence of significantly increased individual effector functions (Ackerman et al., 2016). One individual non-neutralizing antibody activity, antibody dependent cell-mediated cytotoxicity (ADCC) has been linked to EC, particularly in those that are HLA-B*57 − (Lambotte et al., 2013). The role of neutralizing antibodies in natural viral control is not well established. In general, studies have shown that while some EC possess antibodies capable of broad neutralizing activity this is not a general or specific property of this group as a whole.

The present study by Rouers and coworkers found that within EC there was a much higher frequency of HIV Env-specific memory B cell responders compared to patients that have been rendered aviremic through the use of cART (Rouers et al., 2017). Remarkably, this was despite the fact that members of the EC cohort were infected a number of years previously and had presumably controlled viremia to very low levels for an extended period and consequently had experienced minimal ongoing exposure to viral antigen. Equivalent response frequencies to Influenza vaccine antigens were observed in EC and cART recipients, indicating that the reduced HIV-specific B cell frequency observed for the cART group was specific for HIV antigens and did not represent a global disruption of B cell responses. Importantly, it was found that when EC were separated based on their HLA-B*57 allele status, HLA-B*57 + individuals showed a positive correlation between the frequency of HIV Env-specific memory B cells and the breadth of neutralizing activity mediated by sera antibodies, whereas in HLA-B*57 − individuals this correlation was not observed. HIV Env-specific binding titers measured by ELISA were similar for both groups indicating that this difference was qualitative rather than quantitative. HIV Env-specific memory B cell responses in EC were found to be predominantly IgG1, although some subjects also preserved IgG2 and IgG3 responses. HLA-B*57 + and negative EC did not show the preferential preservation of HIV Env-specific B cells expressing a specific isotype. This differs from the potentially protective HIV Gag-specific antibody responses present in sera that have been reported to be more likely to be of the IgG2 isotype in HLA-B*57– controllers (French et al., 2013), although in that study less stringent criteria were used to define controllers compared to those used to define EC in Rouers and coworkers here. The question of whether isotype can influence natural control of HIV infection by antibodies may depend on factors such as the particular antigen targeted and the specific cohort being assessed.

The authors of the present study speculate that early control of viral replication mediated by CD8 + cytotoxic T cells in HLA-B*57 + EC could lead to better CD4 + Tfh cell preservation and that this may be the mechanism behind better preservation of HIV-specific memory B cells. Direct measurement of Tfh and CD8 + T cell subsets in this or other EC cohorts in future studies could help to support this model. In view of the study linking HLA-B*57 − EC to ADCC (Lambotte et al., 2013) it would also be interesting to assess ADCC in the cohort used here. The current study is by necessity a snapshot of the subject's immune system after many years of HIV infection. Earlier events prior to treatment or natural control may shape the development of the immune response, and it would be of great interest to analyze EC and cART recipients at time points closer to the initial infection event where possible. If HIV-specific memory B cell and neutralizing antibody responses contribute to viral control in HLA-B*57 + individuals as suggested by the authors then it would be desirable to measure neutralizing activity against autologous virus. However given the very low viral titers in EC, isolating such virus from samples used in this study would be technically challenging.

Overall the study adds to the growing body of data regarding natural viral control that collectively suggests that multiple immune activities underpin the phenomenon of EC. It is likely that similarly broad immunity will need to be elicited for effective immune-based anti-viral therapeutic or prophylactic vaccine approaches.

## Conflict of Interest

The author declares no conflict of interest relating to the subject matter of this commentary.
